# Comparison of an Emergency Medicine Asynchronous Learning Platform Usage Before and During the COVID-19 Pandemic: Retrospective Analysis Study

**DOI:** 10.2196/58100

**Published:** 2025-02-21

**Authors:** Blake Briggs, Madhuri Mulekar, Hannah Morales, Iltifat Husain

**Affiliations:** 1 Division of Emergency Medicine, Department of Surgery, University of Tennessee Graduate School of Medicine, 1924 Alcoa HighwayKnoxville, TN, 37920, United States, 1 865-696-7221; 2 Department of Mathematics, University of South Alabama, Mobile, AL, United States; 3 Department of Emergency Medicine, Wake Forest School of Medicine, Winston-Salem, NC, United States

**Keywords:** asynchronous learning, medical education, podcast, COVID-19, emergency medicine, online learning, engagement, web-based, online study, online class, videoconferencing, assessment, effectiveness, challenges, knowledge retention, performance, virtual learning, pre-pandemic, post-pandemic

## Abstract

**Background:**

The COVID-19 pandemic challenged medical educators due to social distancing. Podcasts and asynchronous learning platforms help distill medical education in a socially distanced environment. Medical educators interested in providing asynchronous teaching should know how these methods performed during the pandemic.

**Objective:**

The purpose of this study was to assess the level of engagement for an emergency medicine (EM) board review podcast and website platform, before and during the COVID-19 pandemic. We measured engagement via website traffic, including such metrics as visits, bounce rate, unique visitors, and page views. We also evaluated podcast analytics, which included total listeners, engaged listeners, and number of plays.

**Methods:**

Content was designed after the American Board of EM Model, covering only 1 review question per episode. Website traffic and podcast analytics were studied monthly from 2 time periods of 20 months each, before the pandemic (July 11, 2018, to February 31, 2020) and during the pandemic (May 1, 2020, to December 31, 2021). March and April 2020 data were omitted from the analysis due to variations in closure at various domestic and international locations. Results underwent statistical analysis in March 2022.

**Results:**

A total of 132 podcast episodes and 93 handouts were released from July 11, 2018, to December 31, 2021. The mean number of listeners per podcast increased significantly from 2.11 (SD 1.19) to 3.77 (SD 0.76; *t* test, *P*<.001), the mean number engaged per podcast increased from 1.72 (SD 1.00) to 3.09 (SD 0.62; *t* test, *P*<.001), and the mean number of plays per podcast increased from 42.54 (SD 40.66) to 69.23 (SD 17.54; *t* test, *P*=.012). Similarly, the mean number of visits per posting increased from 5.85 (SD 3.28) to 15.39 (SD 3.06; *t* test, *P*<.001), the mean number of unique visitors per posting increased from 3.74 (SD 1.83) to 10.41 (SD 2.33; *t* test, *P*<.001), and the mean number of page views per posting increased from 17.13 (SD 10.63) to 33.32 (SD 7.01; *t* test, *P*<.001). Note that, all measures showed a decrease from November 2021 to December 2021.

**Conclusions:**

During the COVID-19 pandemic, there was an increased engagement for our EM board review podcast and website platform over a long-term period, specifically through website visitors and the number of podcast plays. Medical educators should be aware of the increasing usage of web-based education tools, and that asynchronous learning is favorably viewed by learners. Limitations include the inability to view Spotify (Spotify Technology S.A.) analytics during the study period, and confounding factors like increased popularity of social media inadvertently promoting the podcast.

## Introduction

As the field of medical education evolves, web-based media and digital study tools are finding larger audiences each year [1]. The COVID-19 pandemic dramatically changed the landscape of medical education. Suddenly in March and April 2020, all learning was switched to remote platforms, greatly challenging educators and hastening the switch to web-based media [2-4].

Previous studies have demonstrated that podcasts have positive effects on knowledge retention and test performance [5,6]. Multiple studies have previously been published on the effectiveness of remote learning during the COVID-19 pandemic via remote learning and web-based modules [7,8]. Most recently, 1 study aimed to measure podcast and blog utilization during the early months of the COVID-19 pandemic [9]. This study found an increase in blog page views during the early months of the pandemic, but no statistical change in podcast usage. However, this study had a short measurement period (January to May 2020). In addition, the study made measuring educational content related to COVID-19 a secondary outcome. As asynchronous teaching continues to increase in popularity among students in the wake of the pandemic, medical educators should be curious about the popularity of such materials during a time in which in-person education was severely limited or paused altogether. The purpose of this study was to assess the level of engagement for an emergency medicine (EM) board review podcast and platform, comparing before COVID-19 to during the COVID-19 pandemic over a period of 34 months. Our secondary outcome was to measure important website variables that have previously not been mentioned in medical education literature, especially in the setting of the pandemic. We hypothesized that the pandemic would increase the number of website visitors, page views, and podcast episode plays.

## Methods

### Overview

This retrospective analysis was conducted from March 5, 2022, to April 30, 2022. Data were collected by the study authors from July 11, 2018, when the first podcast episode was released, to December 31, 2021. Emergency Medicine Board Bombs (EMBB) was launched by 2 academic EM physicians in July 2018. The goal of this asynchronous educational platform was to increase first-time pass rate among residents and attendings taking their in-service exam and boards, respectively. EMBB is a peer-reviewed resource and functions at no cost to the learner. EMBB has never been formally assigned to any formal, academic curriculum; its educational platform is entirely free and open access to all learners. The website has podcasts and printable study guides that function as summaries of various common pathologies encountered in the emergency department and on-the-board exams.

### Platform Development

Each podcast episode was structured to quickly cover one multiple-choice question, a discussion of correct and incorrect answers, and the relevant subject matter. Audio-editing was conducted using Apple Garageband, a free service provided to those who own Apple hardware. The podcast was available for free streaming on a designated website, emboardbombs, as well as dedicated podcast platforms (Apple Podcasts, Soundcloud [SoundCloud Global Limited & Co KG], and Spotify [Spotify Technology S.A.]). Questions for each episode were modeled after the American Board of Emergency Medicine (ABEM) certification exam. The Model of the Clinical Practice of Emergency Medicine (EM Model), serves as the basis for ABEM content and was followed in drafting podcast episodes [10]. A peer review process was used to develop multiple-choice questions. Each question was written by an EM physician with an academic appointment and was shared with 2 other academic physicians for review before it was featured on the podcast.

Medical source material was derived from *Tintinalli’s Emergency Medicine* as well as UpToDate and EB Medicine [11-13]. The educational platform was self-funded by the creators and developed on their own time and schedule. No financial support or aid was received. In terms of dedicated time and monetary investment, the cost of equipment and software totaled nearly US $400 annually. In terms of hourly commitment, approximately 5‐10 hours weekly is needed to record, edit, and publish podcast episodes, as well as write and publish study guides. The podcast was not formally added to any curriculum. It was disseminated by word of mouth. No marketing or paid advertising was used.

### Variable Definitions

Podcast analytics were derived from Apple Podcasts Connect which is a free service provided for all Apple Podcast hosts. It provides data on total listeners, engaged listeners, and number of plays [14]. Listeners were defined by Apple as the number of unique devices that played more than 0 seconds of an episode. Engaged listeners were defined as the number of devices that played at least 20 minutes or 40% of an episode within a single session. Of note, pausing or stopping an episode did not count as starting a new session. Number of episode plays was based on the number of unique devices where the play duration is more than 0 seconds. At the time of our data collection during the pandemic, Spotify did not publish podcast statistics, and therefore, their user data could not be obtained.

The website learning platform was hosted on Squarespace. Website traffic analytics were derived from Squarespace, which measured traffic using variables such as website visits, website bounce rate, website unique visitors, and website page views [15]. Visits were defined as the total number of browsing sessions per visitor on the website within a 30-minute period. A browser cookie from Squarespace was used to track views within a 30-minute period. The bounce rate was defined as the number of visitors who navigate away from the website after viewing 1 page. Unique visitors were defined as the total number of new IP addresses that visited the website. Page views were defined as the total number of views across all pages on the website. Page views count the number of times a page is viewed. Furthermore, 1 visit consists of 1 or more pages.

### Data Collection

Website traffic and podcast analytics from July 11, 2018, to February 28, 2020, were compared with those from May 1, 2020, to December 31, 2021. May 1, 2020, was chosen as the transition date because, during March and April 2020, various schools and residency programs began switching to remote learning. As the pandemic evolved, medical schools and graduate medical education sites began suspending in-person rotations. The Accreditation Council for Graduate Medical Education announced in mid-March that all in-person educational activities, meetings, and site visits were to migrate to virtual occurrences only [16]. By the end of April 2020, all nonessential, in-person educational activities had ceased [17].

### Statistical Analysis

All collected data were organized in a Microsoft Excel spreadsheet and analyzed using statistical software JMP Pro 16.0.0 (SAS Institute Inc) in March 2022. All numerical data were summarized using mean and SD. Variations in monthly data from before COVID-19 and during COVID-19 periods were compared using the Levene test, whereas the means per month were compared using a 2-sample *t* test after accounting for differences in variations if any [18,19]. In addition, a nonparametric Mann-Whitney *U* test was also used to compare analytics from 2 time periods. Time series plots were used to study trends in monthly data. A significance level of 0.05 was used to determine the significance of outcomes.

### Ethical Considerations

The Institutional Review Board was approached for ethics approval but reported that the study did not meet the criteria for human candidates research, and therefore, no approval was required.

## Results

During the study period from July 11, 2018, to December 31, 2021, a total of 132 podcast episodes and 93 study guides were created. The first podcast episode was released on July 11, 2018.

From July 11, 2018, to February 28, 2020, 68 episodes were released, along with 30 study guides. From May 1, 2020, to December 31, 2021, 59 podcasts were released, and 53 handouts were published. Note that 5 episodes and 10 handouts were released during March-April 2020, which were also available to learners during the COVID-19 pandemic. This resulted in a total of 225 postings (132 podcasts and 93 handouts) being available to learners during the COVID-19 pandemic (Table 1).

**Table 1.** Number of podcasts, handouts, and total postings before, in-between, and during COVID-19 periods.

Period	Podcasts, n	Handouts, n	Postings, n
Before COVID-19	68	30	98
In-between period	5	10	15
During COVID-19	59	53	112
Total	132	93	225

**Figure 1.**
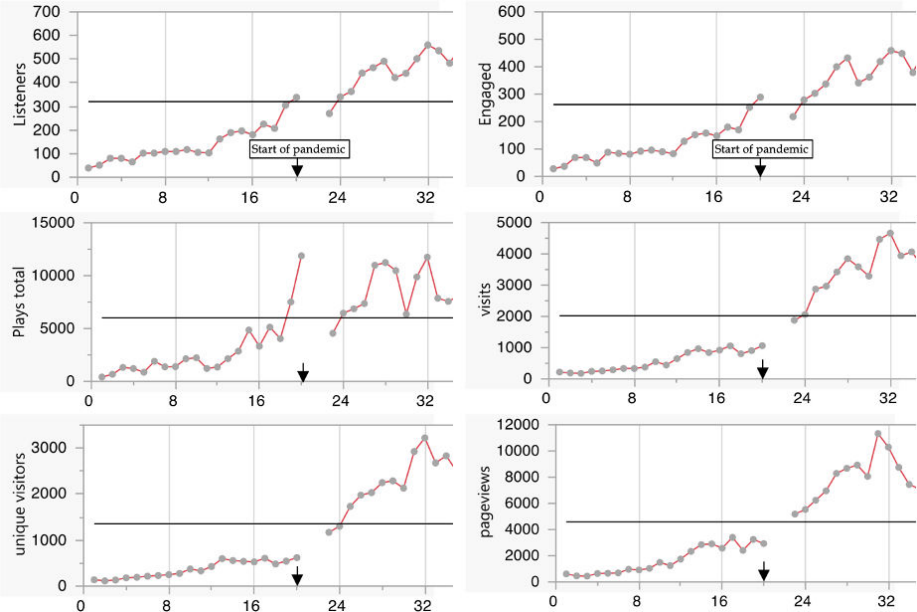
Monthly change in podcast and website visit analytics before COVID-19 and during COVID-19 periods. The arrowhead marks the start of the pandemic.

The time series presented in Figure 1 show month-to-month changes in podcast and website visit analytics before the COVID-19 and during COVID-19 periods and differences in changing patterns. Although higher outcomes were observed during the COVID-19 period in all 6 podcast and website visit measurements compared with before the COVID-19 period, not all changes showed linear patterns of increase. In fact, the number of unique visitors, visits, and page reviews showed decreasing trend after reaching a peak around the middle of the COVID-19 period. However, at the end of the 20-month period, they still remained higher than before the COVID-19 level. During the before the COVID-19 period, number of listeners per month steadily increased from 39 to 338. During the COVID-19 period, it continued to increase, reaching a maximum number of listeners at 672. A similar trend was observed for number engaged per month, increasing from 28 to 289 during the before the COVID-19 period and reaching a maximum of 555 during the COVID-19 period. Although a similar trend was observed for the total number of plays with an increase from 412 to 11,879 during the before the COVID-19 period, a sharp drop was observed during the period of uncertainty (March-April 2020). Again, during the COVID-19 period, total number of plays increased from 4547 to 14,296. Number of visits during the before the COVID-19 period increased from 218 to 1064; there was further increase in the COVID-19 period, reaching 4664 in January 2021. The number of visits started declining thereafter, reaching a low of 1879. The number of unique visitors and page views showed patterns similar to that of the number of visits. The number of unique visitors increased steadily during the before the COVID-19 period from 138 to 620. It increased to 3222 in January 2021 but started declining to a low of 2293. The number of page views also increased steadily during the before the COVID-19 period from 610 to 3405; in the COVID-19 period, it increased to 11,326 in November 2020, only to steadily decrease to a low of 5389 in December 2021. Note that all measures showed a decrease from November 2021 to December 2021.

Comparison of podcast and website visit analytics are presented in Table 2. It shows that regardless of differences in the number of podcasts and handouts available during the 2 time periods, variation in analytics from month to month did not differ significantly during the 2 time periods under study except for bounce rate and number of visitors. Significantly higher variation as measured by SD was observed in bounce rate (0.07 vs 0.05; Levene test, *P*=.036) and number of unique visitors (523.45 vs 179.62; Levene test, *P*=.0049) during COVID-19 pandemic compared with the before the COVID-19 period. Percent increase in mean analytics from before the COVID-19 period to during the COVID-19 period ranged from 24% (bounce rate, 0.55 to 0.30 per 100 postings, n=20) to 539% (unique visitors, 3.74 to 10.41 per posting, n=20) with the mean number of unique visitors showing the highest percent increase and the bounce rate the lowest. The number of visits increased by 504% (5.85 to 15.39 per posting, n=20) whereas the number of listeners, engaged, and total plays each increased by more than 200% (listeners: 2.11 to 3.77 per podcast, n=20; engaged: 1.72 to 3.09 per podcast, n=20; total plays: 42.54 to 69.23 per podcast, n=20). Percent increases in the average monthly analytics indicate considerable increase in visits and usage of podcasts from before COVID-19 to during the COVID-19 period.

**Table 2.** Comparison of podcast and website visit analytics before the COVID-19 and during the COVID-19 periods.

Aspect and period		n	Mean (SD)	Range	*P* value (Levene test)	*P* value (*t* test)	Increase in mean, %
**Listeners**					.54	<.001	247.31
	Before COVID-19	20	143.20 (80.93)	39-338			
	During COVID-19	20	497.35 (99.84)	270-672			
**Engaged listeners**					.48	<.001	247.84
	Before COVID-19	20	117.15 (68.17)	28-289			
	During COVID-19	20	407.50 (82.19)	218-555			
**Number of total episode plays**					.95	<.001	215.88
	Before COVID-19	20	2892.85 (2764.59)	412-11,879			
	During COVID-19	20	9137.80 (2315.19)	4547-14,296			
**Website visits**					.06	<.001	504.3
	Before COVID-19	20	573.20 (321.17)	178-1064			
	During COVID-19	20	3463.85 (689.29)	1879-4664			
**Website bounce rate**					.03	<.001	24.07
	Before COVID-19	20	0.54 (0.05)	46%-62%			
	During COVID-19	20	0.67 (0.07)	52%-75%			
**Website unique visitors**					.004	<.001	538.99
	Before COVID-19	20	366.55 (179.62)	114-620			
	During COVID-19	20	2342.20 (523.45)	1170-3222			
**Website page views**					.27	<.001	346.6
	Before COVID-19	20	1678.60 (1041.70)	443-3405			
	During COVID-19	20	7496.65 (1577.68)	5183-11,326			

Although periods of similar length (ie, 20 months each) were used for comparison, the number of postings available during these 2 periods differed considerably because as new postings were made available, the earlier postings were still available for review for visitors. To account for the differences in the number of postings, analytics were adjusted by computing outcome per posting available. For example, number of listeners per podcast was computed as follows:

- Before COVID-19: # of listeners/podcast = # listeners/68
- During COVID-19: # listeners/podcast = # listeners/132

Note that this accounts for all podcasts that were available to listeners. Before COVID-19 accounts for all podcasts put out during that time and during COVID-19 used all podcasts available, that is, those that were put out before COVID-19, in-between, and during COVID-19 periods. Number of engaged and total plays were adjusted similarly by number of podcasts. Number of visits, unique visitors, and page views were adjusted similarly using all postings (ie, podcasts plus handouts). Bounce rate was adjusted similarly using per 100 postings because rate of per posting resulted in very small numbers and this change from per posting to per 100 postings does not affect the outcome of statistical tests.

Resulting comparisons of outcomes are listed in Table 3, which shows a significant increase in mean rates for all analytics except mean bounce rate per 100 postings from before COVID-19 to during COVID-19. Bounce rate per 100 postings showed a significant decrease from before COVID-19 to during COVID-19 (0.55 to 0.30 per 100 podcasts; *t* test, *P*<.001). Mean number of listeners per podcast increased significantly from 2.11 (SD 1.19) to 3.77 (SD 0.76; *t* test, *P*<.001), mean number engaged per podcast increased from 1.72 (SD 1.00) to 3.09 (SD 0.62; *t* test, *P*<.001), and mean number of plays per podcast increased from 42.54 (SD 40.66) to 69.23 (SD 17.54; *t* test, *P*=.0122). Similarly, mean number of visits per posting increased from 5.85 (SD 3.28) to 15.39 (SD 3.06; *t* test, *P*<.001), mean number of unique visitors per posting increased from 3.74 (SD 1.83) to 10.41 (SD 2.33; *t* test, *P*<.001); and mean number of page views per posting increased from 17.13 (SD 10.63) to 33.32 (SD 7.01; *t* test, *P*<.001). Even nonparametric comparisons using Mann-Whitney *U* test gave the same results.

**Table 3.** Comparison of podcast and website visit analytics rates per posting available to viewers before COVID-19 and during COVID-19 periods.

Aspect and period		n	Mean (SD)	Range	Median (IQR)	*P* value (*t* test)	*P* value (Mann Whitney *U* test)
**Listeners per podcast**						<.001	<.001
	Before COVID-19	20	2.11 (1.19)	0.57-4.97	1.60 (1.26-2.86)		
	During COVID-19	20	3.77 (0.76)	2.05-5.09	3.83 (3.34-4.27)		
**Engaged per podcast**						<.001	<.001
	Before COVID-19	20	1.72 (1.00)	0.41-4.25	1.34 (1.06-2.30)		
	During COVID-19	20	3.09 (0.62)	1.65-4.20	3.22 (2.62-3.47)		
**Number of total episode plays per podcast**						.0122	<.001
	Before COVID-19	20	42.54 (40.66)	6.06-174.69	29.71 (18.36-56.81)		
	During COVID-19	20	69.23 (17.54)	34.45-108.30	69.95 (56.13-81.99)		
**Website visits per posting**						<.001	<.001
	Before COVID-19	20	5.85 (3.28)	1.82-10.86	5.07 (2.68-9.08)		
	During COVID-19	20	15.39 (3.06)	8.35-20.73	15.64 (13.80-17.41)		
**Website bounce rate per 100 postings**						<.001	<.001
	Before COVID-19	20	0.55 (0.05)	0.47-0.63	0.54 (0.51-0.59)		
	During COVID-19	20	0.30 (0.03)	0.23-0.33	0.30 (0.27-0.32)		
**Website unique visitors per posting**						<.001	<.001
	Before COVID-19	20	3.74 (1.83)	1.16-6.33	3.60 (2.03-5.53)		
	During COVID-19	20	10.41 (2.33)	5.20-14.32	10.65 (9.14-11.84)		
**Website page views per posting**						<.001	<.001
	Before COVID-19	20	17.13 (10.63)	4.520-34.745	13.98 (6.85-28.35)		
	During COVID-19	20	33.32 (7.01)	23.036-50.338	32.49 (28.39-38.13)		

## Discussion

### Principal Findings

The results demonstrate that our online EM board review podcast and platform experienced significantly increased levels of engagement during the COVID-19 pandemic. Our learning platform included multiple media, such as PDF study guides, video and picture-based modules, and online question banks. The aim was for the podcast and handouts to be integrated into an asynchronous study plan, as the platform provided easy accessibility and use.

### Implication of Findings

The COVID-19 pandemic disrupted medical education, forcing learners in both medical school and residency to navigate vast amounts of information, largely in isolation. This shift from interactive, in-person learning raised concerns about students overextending themselves, leading to only a surface-level understanding of the material. One study comparing first and second-year medical student education during the pandemic highlighted the importance of face-to-face learning, finding that the first-year medical students in isolation performed worse than the previous year’s first-year medical students [20]. Another retrospective study performed at the University of Hawaii Burn School of Medicine demonstrated that fourth-year medical students who were enrolled during the pandemic displayed improved note-taking with a 9-point increase in exam scores, yet worse physical examinations in their standardized patient encounters with a 12-point average decrease in scores [21].

In response, many innovative educational tools have emerged to attempt to provide asynchronous learning. Online resources like the one in this study are unique. Diverse topics are integrated into a single, cost-effective, and efficient platform, with podcast episodes <20 minutes, as well as downloadable PDF handouts. This model is beneficial for both visual and auditory learners.

While other learning platforms were not analyzed during this study period, valuable information was collected from this study’s podcast. EMBB offers a humanistic aspect to learning with the dual physician hosts, pertinent banter, and narrative medicine aspect, of which may anthropomorphize the learning despite pandemic isolation.

### Comparison With the Literature

Podcasts have been welcomed by those looking for a nontraditional method of learning in recent years, most notably those practicing in EM, where it is the most represented specialty that regularly hosts podcasts [22-24]. A survey in 2014 showed EM residents devote more time to podcasts than journals, citing podcasts as “the most beneficial” for education [22]. In another large survey, 80% of EM residents had listened to medical podcasts at least once [25].

Traditional lectures continue to be replaced by various digital teaching methods and this was hastened by the arrival of COVID-19. Podcasts’ major benefit is their customization to fit learner’s educational goals as well as time constraints, allowing users to optimize their study goals while balancing work and private life.

### Feasibility of Implementation

In terms of feasibility, the podcast required a dedicated amount of time and monetary investment. The cost of standard microphones, basic recording software, and a website to host the podcast required approximately US $300 to 400 annually. As discussed in the methods section, the hourly commitment was close to 5‐10 hours weekly.

### Next Steps

A review of “Learning Through Listening: A Scoping Review of Podcast Use in Medical Education” examines podcasts for learning across many specialties, most often referencing anesthesia, with some reference to EM [26]. The data cited an increase in retention of information pre- and posttest for medical students, who are not specialized in EM compared with the level of a resident or attending physician. The review briefly mentions a podcast that improved EM in-training exam scores and a podcast that reportedly worsened in-training exam scores. The data gleaned from this study are of interest, but due to varied in-training exam scores, a comparative study is needed that examines test performance matching which podcast was used most for learning. Another future area of study will be to observe if the effects of the COVID-19 pandemic on asynchronous web-based learning are long-term.

### Limitations

Our study is limited in generalizability due to it only measuring one specific podcast and website platform. A restricted sample size is one limitation of this study. Spotify and Android (Google) do not publish podcast statistics nor track individual usage, and therefore user data from both these platforms could not be obtained. According to Reuters in a survey of 2012 listeners, 20% used Apple Podcasts as their app of choice from 2019‐2020, which is the second largest market share [27]. Previous studies have used podcast episode downloads as a metric for engagement. Despite the appeal of using number of downloads as a measurement, accurate analytics are difficult to obtain and fraught with bias. Downloads are defined differently depending on the podcast host. In addition, there have been reports that these numbers can be unreliable due to bot traffic and there can be manipulation of download data by hosts [28,29].

Another limitation is association versus causation. Given the retrospective study design and nature of COVID-19, it is difficult to completely credit the pandemic for increased podcast engagement. Confounding variables could also be a limitation, such as increased usage of social media during quarantine resulting in better promotion of the podcast and website.

One potential confounding variable was the launch of a procedural module in May 2020. This web-based learning instruction was an airway module, with recorded intubation videos and a pre- and postassessment. However, when reviewing website analytics, this was not a frequently viewed page on the website, accounting for only 2.59% of total website page views. It cannot entirely account for the sudden increase in website visitors and podcast listeners. Thus, in this study, we can only establish differences observed in analytics between 2 time periods.

No quantitative data were tracked regarding listener exam performance, in particular in-training or board examinations. The purpose of this study was to assess the level of engagement for an EM board review podcast and website platform, before and during the COVID-19 pandemic. Future research should be aimed at assessing whether this educational intervention is an effective form of test preparation.

### Conclusion

During the COVID-19 pandemic, there was an accelerated level of engagement for our EM board review podcast and website platform over a long-term period. This educational platform is a feasible, low-cost asynchronous study tool. Medical educators should be aware of the increasing usage of web-based education tools, and that asynchronous learning is favorably viewed by learners.

## Abbreviations

ABEM: American Board of Emergency Medicine
EM: emergency medicine
EMBB: Emergency Medicine Board Bombs
